# Rapid uniaxial actuation of layered bacterial cellulose/poly(*N*-isopropylacrylamide) composite hydrogel with high mechanical strength†

**DOI:** 10.1039/c8ra01639a

**Published:** 2018-04-03

**Authors:** Qidong Wang, Taka-Aki Asoh, Hiroshi Uyama

**Affiliations:** Department of Applied Chemistry, Graduate School of Engineering, Osaka University 2-1 Yamadaoka Suita Osaka 565-0871 Japan uyama@chem.eng.osaka-u.ac.jp

## Abstract

This study deals with the unique morphology and properties of methylene diphenyl diisocyanate (MDI)-modified bacterial cellulose/poly(*N*-isopropylacrylamide) (BC/PNIPAAm) composite hydrogel prepared by *in situ* polymerization method. The influence of the molar ratio of MDI/glucose unit of BC on the properties of the resulting hydrogel was investigated. Scanning electron microscopic analysis revealed that after the MDI modification the BC/PNIPAAm hydrogel could preserve the unique layered (known as anisotropic) structure. The mechanical property evaluated by stress–strain test was significantly enhanced when compared to that of neat PNIPAAm hydrogel, due to the presence of the BC matrix as well as the MDI modification. Based on the deswelling behaviors, the BC/PNIPAAm hydrogel exhibited improved and controlled responsive rate when compared with neat PNIPAAm hydrogel. Furthermore, the anisotropic thermo-sensitive property was proved by temperature-responsive test with the fact that the composite hydrogel could only deswell and swell in the axial perpendicular to the layers. Along with desired recyclability, the present composite hydrogel may have an application as artificial muscles.

## Abstract 2

Anisotropic thermo-sensitive composite hydrogel based on bacterial cellulose was prepared by *in situ* polymerization, which could swell and deswell uniaxially.

## Graphical abstract

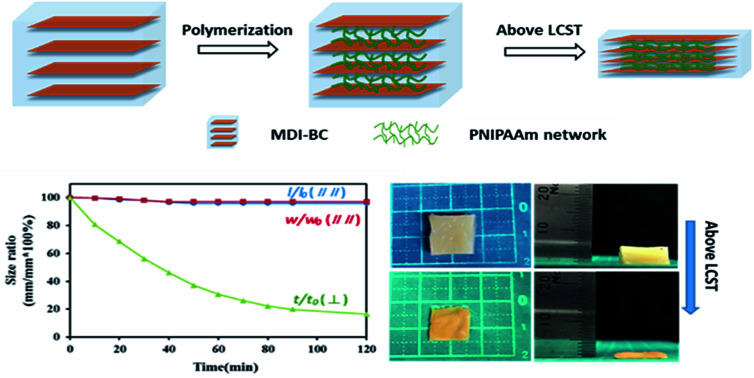

## Introduction

Anisotropic swelling and deswelling of hydrogels are essential for their utilization in biomedical fields. Many biological systems have well-defined anisotropic structures, which is beneficial to carry out particular functions, including mass transport, surface lubrication, and force generation.^1^ As a representative example, in a muscle sarcomere, actin and myosin show an anisotropic arrangement, which contributes the smooth motion of muscle fibers and muscle contraction in one direction while keeping other the direction constant.^2–4^ Unfortunately, synthesized hydrogels are usually isotropic due to their preparation methods, which leads to homogeneous movement in response to external stimuli. Thus their applications in this field are greatly limited.

Poly(*N*-isopropylacrylamide) (PNIPAAm) is one of the most investigated polymers for preparing hydrogel actuators. It undergoes reversible lower critical solution temperature (LCST) phase transition from a swollen hydrated state to a shrunken dehydrated state, which is the result of the competition between the hydrophilic amide and hydrophobic isopropyl moieties in the polymer side-chain.^5–7^ LCST of PNIPAAm hydrogel is close to body temperature and relatively insensitive to other environmental conditions such as pH and light, which makes PNIPAAm suitable for biomedical application.^8^ Previously, anisotropic movement of PNIPAAm-based hydrogels has been successfully achieved.^9–13^ However, the volume changes of the PNIPAAm hydrogels are known to proceed very slowly,^14,15^ which indicates unsatisfactory responsive rate. Fast actuation in response to temperature change is often important for smart hydrogels. Furthermore, like other hydrogels, common PNIPAAm hydrogels possess poor mechanical properties.

Bacterial cellulose (BC) is a promising candidate as polymer matrix to enhance PNIPAAm hydrogels. Compared to widely used plant-derived cellulose, BC enjoys its distinguishing advantages such as high purity, high porosity and high water content.^16–18^ Along with good biodegradability and biocompatibility, BC becomes an ideal choice in biomedical and biotechnological fields. It's potential applications are wound dressing, tissue regeneration, skin substitutes^19–22^ and drug delivery system.^23,24^ The high crystallinity of BC results in excellent mechanical strength of high Young's modulus, tensile and compressive strength,^25,26^ making it act as ideal reinforcing element in polymer matrix. Moreover, BC with layered structure can be prepared by static culture of certain bacteria.^25,27^ This kind of anisotropic structure brings about mechanical anisotropy to BC.^28^ In spite of these outstanding features, pristine BC lacks certain functions and properties (*i.e.*, stimuli–responsive property), which limits its applications in various fields. Therefore, synthesis of BC composites has been conducted to address these limitations. Many researchers focus on the preparation of BC-based functional composites with other polymers or inorganic materials to enlarge the potential applications of BC.^29–31^

**Fig. 1:**
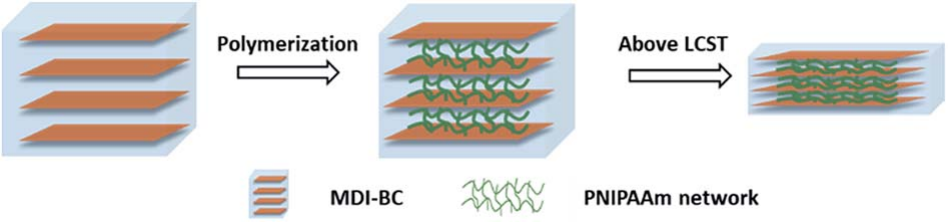
Schematic illustration of complex formation of MDI-BC/PNIPAAm and uniaxial deswelling above LCST.

In this study, in order to overcome the shortcomings of BC and PNIPAAm hydrogels, a 4,4′-methylenediphenyl diisocyanate (MDI)-modified BC/PNIPAAm composite hydrogel was prepared by a simple *in situ* polymerization method. With the layered structure, the composite hydrogel would show the desired anisotropic thermo-sensitivity (Fig. 1). The morphological, thermal and mechanical properties of the resultant product was investigated by scanning electron microscopy (SEM), differential scanning calorimetry (DSC), and compressive stress–strain measurements to reveal their structure, LCST behavior and physical strength. The temperature dependences of the swelling ratio and deswelling behaviors of the hydrogel were characterized. In particular, the size changes of the hydrogel in three dimensions were carefully studied to confirm the anisotropic thermo-sensitivity of the present composite hydrogel. By the combination of MDI-modified BC and PNIPAAm, the composite hydrogel is aimed to have uniaxial deswelling–swelling (so-called anisotropic thermo-sensitivity), enhanced mechanical strength as well as fast response rate to temperature change.

## Experimental

### Materials

NIPAAm, ammonium persulfate (APS), MDI were purchased from Tokyo Chemical Industry Co., Ltd., Japan. *N*,*N*′-Methylenebisacrylamide (MBAAm) and *N*,*N*,*N*′,*N*′-tetramethyl-ethylenediamine (TEMED) were obtained from Wako Pure Chemical Industries, Ltd., Japan. Triethylamine (TEA) and acetone were purchased from Nacalai Tesque, Inc., Japan. BC used in this experiment was prepared according to our previous paper.^32^

### Preparation of MDI-modified BC hydrogel

Medium of BC hydrogel was first exchanged with dehydrated acetone by immersing the BC hydrogel disc (1.5 g) into an excess of dehydrated acetone for 8 h under gentle shaking. Solvent replacement was repeated at least 3 times. A certain amount of MDI was then added to the acetone solution (20 mL) in the presence of the BC organogel, followed by the addition of TEA (76 μL). The molar ratio between MDI and the glucose unit of BC was set as 0.2 : 1, 1 : 1, and 2 : 1. The mixture was shaken at 25 °C for 3 h before it was kept at 50 °C for 48 h to form the MDI-modified BC organogel. The MDI-modified BC hydrogel was obtained by washing with acetone and deionized water.

### Synthesis of MDI-modified BC/PNIPAAm composite hydrogel

NIPAAm monomer (1.0 g) and MBAAm (0.010 g) were first dissolved in deionized water (7.4 mL) to make the monomer solution. The MDI-modified BC disc (∼1.6 g) was then immersed in the above solution for 12 h. APS (0.010 g) and TEMED (6.0 μL) were then added to start the polymerization. The whole weight of all the reagents and solvent was 10 g. After 12 h, the formed BC/PNIPAAm hydrogel was washed by deionized water. The MDI-modified BC/PNIPAAm composite hydrogels were coded as COM1, COM2 and COM3 according to the molar ratio between MDI and the glucose unit of BC from 0.2 : 1 to 2 : 1. The BC/PNIPAAm hydrogel without the MDI modification, as a control, was coded as COM0.

### Characterization

Fourier transform infrared spectroscopic analysis (FT-IR) was performed in an attenuated total reflectance (ATR) mode by a Nicolet iS5 Spectrometer (Thermo Fisher Scientific Inc., USA). SEM images were obtained on a HITACHI S-3500 instrument (Hitachi Co., Japan). The samples were lyophilized before the SEM observation. A compressive test was performed by a universal testing machine (EZ Graph, SHIMADZU, Japan). The samples (*c.a.* 20 mm in diameter and 10 mm in height) were fixed between two plates with compressive speed of 60 mm min^−1^ to obtain a typical stress–strain curve. LCST of the hydrogel samples was determined by a differential scanning calorimeter (EXSTAR 6000 DSC, Hitachi High-Tech Science Co., Japan). The thermal analysis was performed from 20 °C to 60 °C at the heating rate of 5 °C min^−1^ under nitrogen. The onset point of the endothermal peak was used to determine the LCST.^33,34^

### Thermo-sensitive property measurement

For the equilibrium swelling ratio (ESR) measurement, the hydrogel samples were swollen in distilled water over a temperature range from 20 to 55 °C, covering the LCST range of PNIPAAm hydrogel. The gravimetric method was employed; the samples were immersed in distilled water at predetermined temperature for 24 h to reach swelling equilibrium, and they were taken out and weighed after removing the excess water. ESR was calculated as follows:

1 Swelling ratio = (*W*_s_ − *W*_d_)/*W*_d_

where *W*_s_ and *W*_d_ are the weight of the swollen and dried hydrogels, respectively.

The deswelling behaviors of the hydrogel were studied at 50 °C (above LCST) gravimetrically. At regular time intervals, the samples were taken out and weighed after removing the excess water. Water retention is defined as follows:

2 Water retention = [(*W*_t_ − *W*_d_)/(*W*_e_ − *W*_d_)] × 100

where *W*_t_ is the weight of the hydrogel at a determined time at 50 °C, *W*_e_ is the weight of the hydrogel at equilibrated swelling at 20 °C, and other symbols are the same as defined above.

For the anisotropic thermo-sensitive property, the samples were immersed in water at 50 °C. *l*/*l*_0_ and *w*/*w*_0_ were shrinking (or deswelling) ratios parallel to the layer structure of BC/PNIPAAm hydrogel, and *t*/*t*_0_ was that perpendicular, where *l*_0_, *w*_0_, *t*_0_ were the length, width and thickness of the gel samples at equilibrium state in 20 °C solution and *l*, *w*, *t* were those at determined time at 50 °C in water solution. The length, width and thickness of the hydrogel samples were measured by a caliper.

## Results and discussion

The dry weights of the samples during each step were measured and summarized in Table 1. It can be calculated that the molar ratios between MDI and the glucose unit of BC (after reaction) were 0.19 : 1, 0.98 : 1 and 1.77 : 1 for COM1, COM2 and COM3, respectively. Similarly, the weight ratio of PNIPAAm in the final dried products could be calculated as 89%, 86%, 73% and 60% for COM0, COM1, COM2 and COM3, respectively, indicating successfully prepared PNIPAAm hydrogels in the presence of a hydrophobically modified BC.

**Table 1:** Dry weights of all the samples after each step^a^

	COM0	COM1	COM2	COM3
MDI-BC (mg)	—	22	43	64
MDI : glucose unit of BC (mol : mol)	—	0.19 : 1	0.98 : 1	1.77 : 1
BC/PNIPAAm or MDI-BC/PNIPAAm (mg)	160	161	161	161
Weight ratio of PNIPAAm (wt%)	89%	86%	73%	60%

a Dry weights of BC in all the samples were 17 mg.

MDI-modified BC/PNIPAAm composite hydrogels were also studied by FT-IR spectra (Fig. S1†). Despite the difference in the MDI/glucose unit molar ratios, all the samples showed very similar FT-IR spectra. A sharp absorption peak at 3300 cm^−1^ is assigned to hydroxyl groups of BC. The samples had peaks at 1640 and 1540 cm^−1^ which are referred to the amide I and amide II of PNIPAAm, respectively. Notice that there was no obvious difference between the spectrum of COM0 hydrogel and those of COM1, COM2, COM3. This is probably due to the reason that the IR spectrum of the urethane group derived from MDI is similar to that of PNIPAAm with the amide group.

**Fig. 2:**
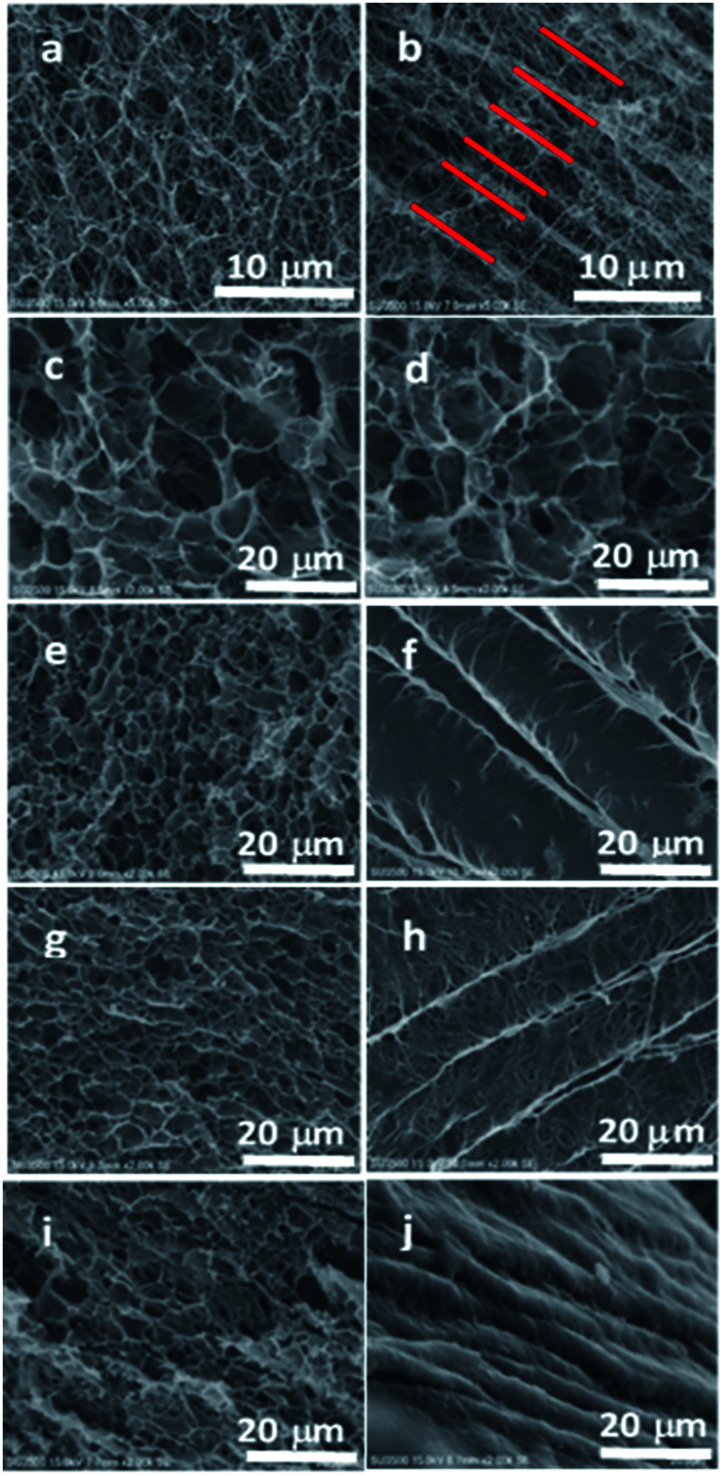
SEM images of pure BC (a and b), COM0 (c and d), COM1 (e and f), COM2 (g and h), COM3 (i and j). Horizontal and vertical images are on the left and right columns, respectively. Red bars indicate the layered structure of BC.

According to the SEM images of pure BC hydrogel (Fig. 2a and b), the 3D network structure of BC was confirmed in the horizontal image while it showed an excellent layered structure in the vertical direction, indicating the anisotropic structure of BC. However, the BC/PNIPAAm hydrogel (COM0) lost this layered structure (Fig. 2d), resulting in porous morphology. When BC was cross-linked by MDI before the polymerization of PNIPAAm, the layered structure in the vertical direction could preserve to some extent (Fig. 2f, h and j) while the horizontal images displayed the 3D network structure. The more the MDI ratio, the better the layered structure inside the hydrogel remained. Thus the average thickness of the layers decreased with increasing the MDI ratio and the average thickness of COM3 was close to that of pure BC. The SEM observation indicates that the anisotropic composite hydrogels were successfully prepared. This unique structure was believed to endow special properties to the hydrogels, which would be mentioned below.

**Fig. 3:**
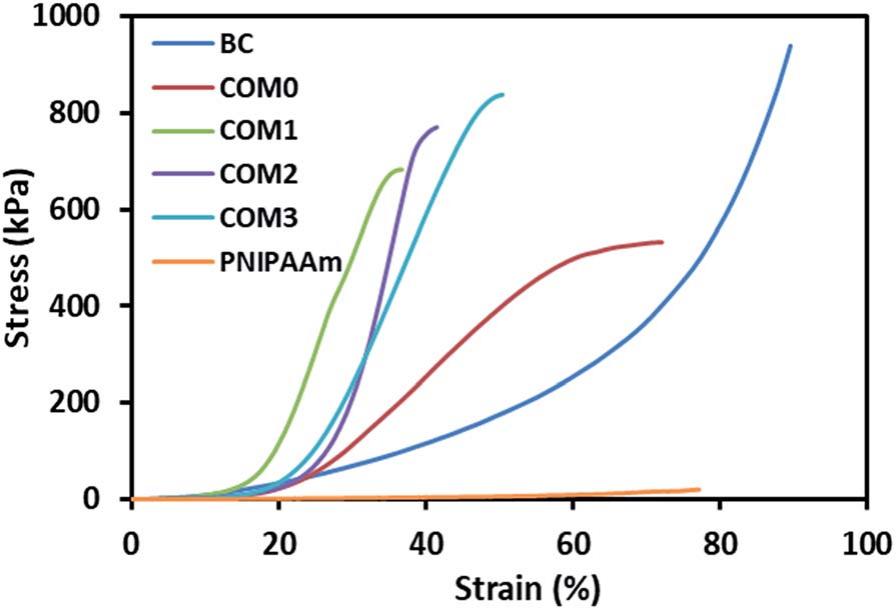
Stress–strain curves of BC/PNIPAAm composite hydrogels with different MDI ratio, BC and PNIPAAm hydrogels under compression.

Fig. 3 shows the stress–strain curves of the composite hydrogels including pure BC in the vertical direction (perpendicular to the layered structure), whereas data obtained from these tests can also be found in Table 2. The data prove that the introduction of BC and MDI-modified BC would greatly improve the mechanical property of PNIPAAm hydrogel. The maximum compressive strength of COM0 was 532 kPa whereas that of PNIPAAm hydrogel was 20 kPa. With the increase of the MDI/glucose unit ratio, the compressive strength increased gradually. COM3 had the largest compressive strength of 838 kPa, which was about 40 times of that of the PNIPAAm hydrogel. This significant improvement of mechanical strength is believed to be beneficial to various applications. The SEM images show that the composite hydrogel with the higher MDI/glucose unit ratio has the smaller layer thickness, making the sample more rigid. As a result, the MDI-modified BC/PNIPAAm hydrogel with the higher MDI/glucose unit ratio would have better mechanical strength.

**Table 2:** Mechanical properties of BC, PNIPAAm and BC/PNIPAAm composite hydrogels with different MDI ratio

Sample	Compressive strength (kPa)	Strain at break (%)
BC	938	89
COM0	532	72
COM1	682	36
COM2	770	41
COM3	838	50
PNIPAAm	20	77

The LCST of the series of the hydrogel samples were examined by DSC (Fig. S2†). The onset temperature of endotherm was referred as LCST. Obviously, all the samples showed almost the same LCST at about 32 °C, which matched well with the LCST of PNIPAAm hydrogel. From these data, the BC or MDI-modified BC hydrogel was found to have little impact on LCST of PNIPAAm, indicating that the networks of BC (or MDI-BC) and PNIPAAm are chemically identical and no reaction occurred between them. It is well known that LCST is the point where the hydrophobic interaction of the isopropyl group of PNIPAAm outweighs the hydrophilic nature of the amide group in the pendant group, forcing water out of the hydrogel.^35^ The subsequent research and applications are mainly based on this LCST of the composite hydrogel.

**Fig. 4:**
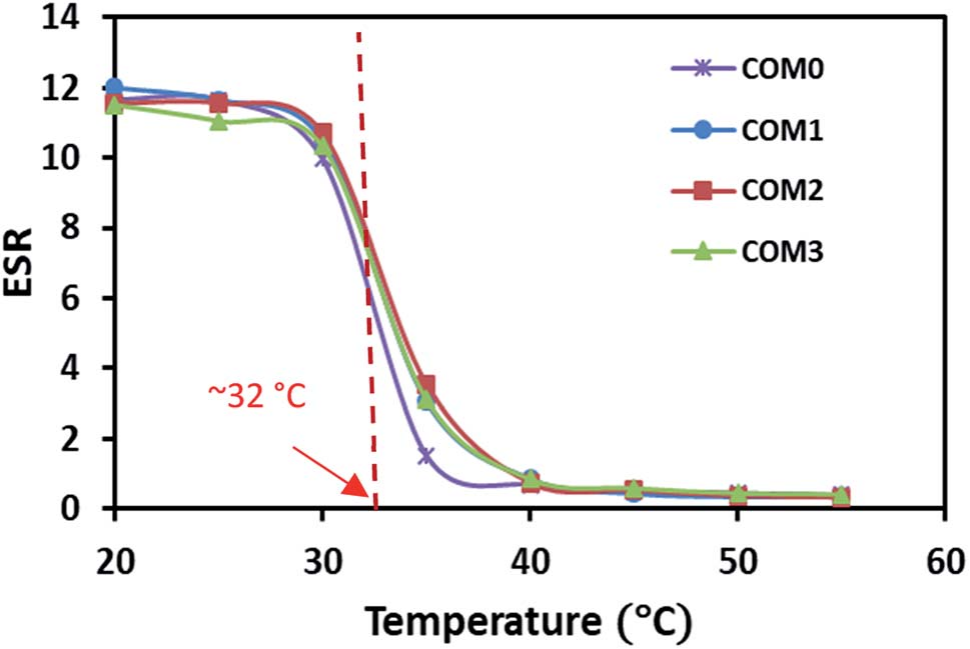
ESR of the composite hydrogels in the temperature range from 20 °C to 55 °C.

The temperature dependence of ESR is shown in Fig. 4. The swelling data here showed that all the samples had similar classical thermo-responsive profile. The ESR of all the hydrogels decreased dramatically toward their LCST and had the sharpest decrease around 32 °C where the phase separation occurred. Above the LCST, the hydrogel samples showed almost the same level of ESR regardless of the MDI ratio difference. The LCST from the ESR observation is in good agreement with the thermal data of the DSC study.

**Fig. 5:**
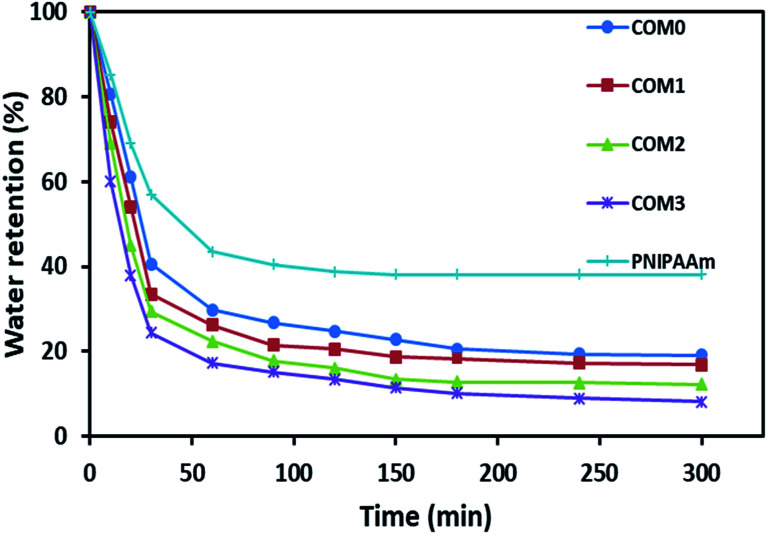
Deswelling behaviors of the composite hydrogel samples at 50 °C.

Fig. 5 exhibits the deswelling behaviors of the composite hydrogels in water at 50 °C (above LCST). After 20 h, all the five hydrogels including PNIPAAm had a similar water retention of around 3–4% (data not shown in Fig. 5). However, the composite hydrogels showed the relatively faster response to this temperature in the initial 60 min, *i.e.*, COM3 lost about 40% water in the first 10 min whereas pure PNIPAAm hydrogel only lost less than 15% water in the same time period. About 76% water was freed from COM3 in 30 min whereas PNIPAAm showed only 43% water release in the same time frame. The order of water lose rate within 30 min was COM3 > COM2 > COM1 > COM0 > PNIPAAm. The composition between BC and PNIPAAm as well as the MDI modification significantly improved the responsive rate of the hydrogel. Generally, PNIPAAm-based hydrogels present slow responding property to temperature. For example, Okano *et al.* reported that only 15% volume shrinkage was observed for a PNIPAAm-based hydrogel disk for 60 min on heating from 10 to 40 °C.^14^ Up to now, three main kinds of strategies have been developed to improve the response rate of PNIPAAm-based hydrogels as follows: (1) diminishing the dimension of hydrogels, (2) generating porous structures of hydrogels, and (3) chemically modifying polymeric networks of hydrogels.^36^ In our case, the size of PNIPAAm gel in the BC/PNIPAAm composite was reduced mainly due to the layered BC matrix, leading to the rapid response to temperature change. On the other hand, the PNIPAAm-based hydrogels usually form dense hydrophobic layers on outmost surface – so-called skin layers above LCST,^37^ which results in slow volume change. BC with high hydrophilicity in the BC/PNIPAAm composite hydrogels may inhibit the formation of the skin layers, which would induce the increase in the response rate of the composite hydrogel.

**Fig. 6:**
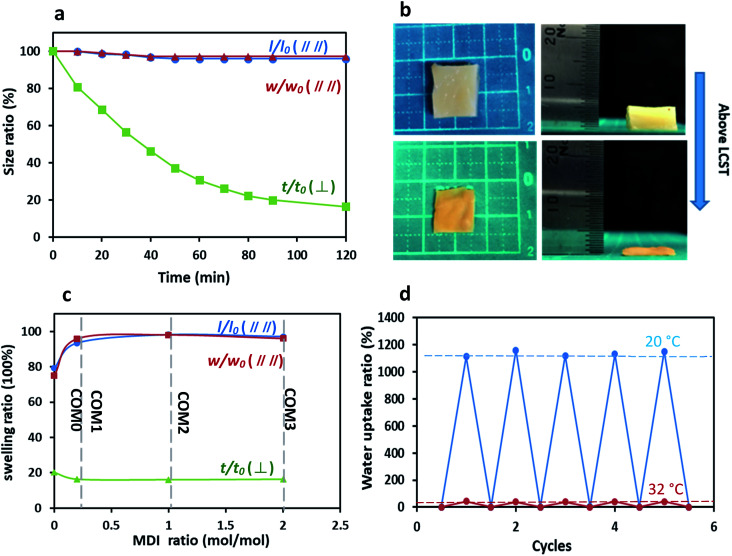
(a) Length (*l*), width (*w*) and thickness (*t*) change of COM3 above LCST with time; (b) photos of uniaxial deswelling of COM3 above LCST for 120 min. Left and right images indicate top and side views, respectively; (c) anisotropic deswelling of the hydrogels with different MDI ratios above the LCST; (d) repeatable swelling behavior of COM3 gel below or above LCST.

Commonly, PNIPAAm-based hydrogels have a homogeneous structure, resulting in an isotropic thermo-sensitive property. On the other hand, the anisotropic structure of the present MDI-modified BC/PNIPAAm hydrogels as shown in the SEM images influences the thermo-sensitivity. As shown in Fig. 6a, COM3 clearly exhibited the uniaxial deswelling behavior in response to temperature change. The thickness of the hydrogel decreased gradually whereas the length and width remained almost unchanged. The photos of uniaxial deswelling of COM3 above LCST are shown in Fig. 6b. The equilibrium deswelling of the hydrogels with different MDI ratios above the LCST is concluded in Fig. 6c. In the direction perpendicular to the layered structure (vertical), COM0, COM1, COM2 and COM3 shared a similar thickness deswelling ratio (*t*/*t*_0_) of about 20%. However, the deswelling ratio parallel to the layered structure (horizontal) gave the different result: *l*/*l*_0_ and *w*/*w*_0_ of COM0 were less than 80% whereas those of COM1, COM2 and COM3 were more than 95%. In the present composite hydrogel, the PNIPAAm network could swell freely only in the direction perpendicular to the BC layers whereas in other two directions (parallel to the layers), the swelling was almost completely restricted by the rigid BC layers, resulting in the uniaxial deswelling–swelling. Once deswelling above LCST, the BC/PNIPAAm gel could swell back to its original hydrogel state below LCST. This process was reversible and easily controlled by temperature change. The SEM images of the dried composite gel (COM3) were shown in Fig. S3.† Visibly, the BC fibrils arranged randomly in the horizontal direction whereas in the vertical direction the sample showed the excellent layered structure. Therefore, the BC fibrils in the dried composite gel are arranged in an anisotropic manner. Fig. 6d shows the repeatable swelling behavior of dried COM3 below or above LCST. It hardly absorbed any water at 50 °C because PNIPAAm showed hydrophobicity above LCST. However, at 20 °C the dried COM3 swelled well and absorbed a large amount of water (>1000% of the dried gel weight) to recover back to its original hydrogel state. Moreover, the water uptake ability of COM3 almost remained unchanged below or above LCST after many cycles, indicating the desired recyclability of the present composite hydrogel.

## Conclusions

In this study, we developed a novel MDI-modified BC/PNIPAAm composite hydrogel by *in situ* polymerization and elucidated the changes in morphology, mechanical properties, response rate to temperature, and thermo-sensitive properties. Only the MDI-modified BC/PNIPAAm composite hydrogel exhibited an anisotropic layered structure in which the average layer thickness of the hydrogel decreased with the increase of the MDI/glucose molar ratio. It was clearly observed that the composition between BC and PNIPAAm as well as MDI modification contributed to the reinforcement of PNIPAAm gel and the present hydrogel exhibited 40 times higher compressive strength than neat PNIPAAm gel. Besides, the composite hydrogel showed improved response rate to temperature which depended on the MDI/glucose molar ratio. This controllable response rate is significant for future practical applications. Furthermore, the anisotropic thermo-sensitivity of the composite hydrogel was revealed due to the reason that the gel only swelled and deswelled perpendicular to the layers uniaxially. The unique temperature-responsive property as well as enhanced physical strength and adjustable response rate makes the MDI-modified BC/PNIPAAm composite hydrogel a promising choice in biomedical fields such as artificial muscles.

## Conflicts of interest

There are no conflicts to declare.

## Supplementary Material

RA-008-C8RA01639A-s001
