# Supplementary material for: Rapid uniaxial actuation of layered bacterial cellulose/poly(N-isopropylacrylamide) composite hydrogel with high mechanical strength
Source: RSC Adv. 2018 Apr 3;8(23):12608–13. doi: 10.1039/c8ra01639a (PMC9079606; doi:10.1039/c8ra01639a)
Supplement: RA-008-C8RA01639A-s001 [file RA-008-C8RA01639A-s001.pdf]

Electronic Supplementary Material (ESI) for RSC Advances.
This journal is © The Royal Society of Chemistry 2018

Supporting information

Rapid uniaxial actuation of layered bacterial cellulose/ poly(*N*-isopropylacrylamide) composite hydrogel with high mechanical strength

Qidong Wang, *Taka-Aki Asoh*, and *Hiroshi Uyama**

Department of Applied Chemistry, Graduate School of Engineering, Osaka University

2-1 Yamadaoka, Suita, Osaka 565-0871, JAPAN

Phone: +81-6-6879-7364, Fax: +81-6-6879-7367

e-mail: uyama@chem.eng.osaka-u.ac.jp

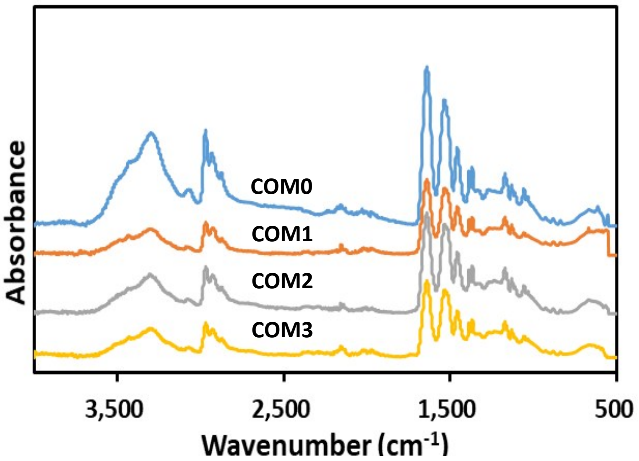

Fig. S1 FT-IR spectra of BC/PNIPAAm hydrogel with different MDI ratios.

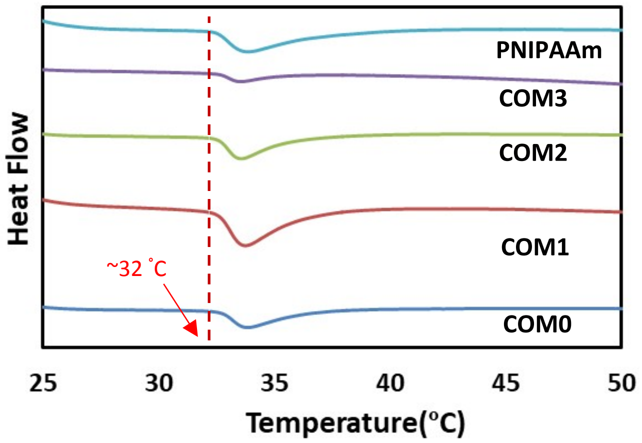

Fig. S2 DSC traces of swollen hydrogel samples

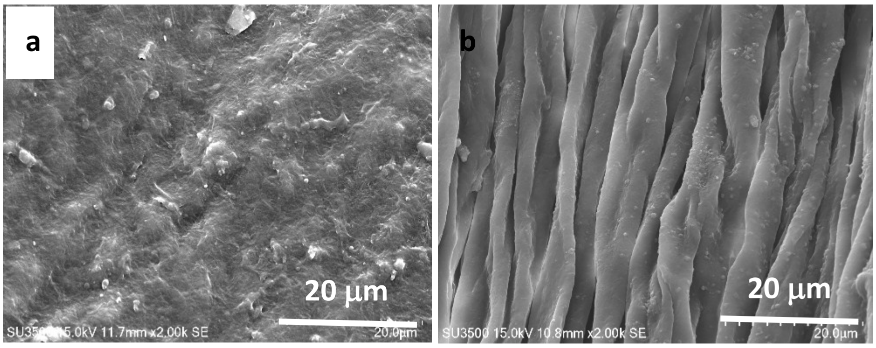

Fig. S3 SEM images of dried COM3: (a) horizontal and (b) vertical images are on the left and right columns, respectively.
